# *Monascus* spp. and Their Relative Products

**DOI:** 10.3390/jof12090672

**Published:** 2026-09-07

**Authors:** Fusheng Chen, Yanchun Shao

**Affiliations:** 1 College of Food Science and Technology, Huazhong Agricultural University, Wuhan 430070, China; yanchunshao@mail.hzau.edu.cn; 2 Hubei International Scientific and Technological Cooperation Base of Traditional Fermented Foods, Huazhong Agricultural University, Wuhan 430070, China

*Monascus* spp. are a group of filamentous fungi with nearly 2000 years of application history in Asia, particularly in China, for producing herbal and edible products. These fungi can produce a rich array of beneficial secondary metabolites, such as natural edible *Monascus* pigments (MPs) and the lipid-lowering component monacolin K (MK). However, some *Monascus* strains may also produce the mycotoxin citrinin (CIT), which poses potential food safety risks. It is important to note that not all *Monascus* strains can produce citrinin, as some *Monascus* genomes lack the gene cluster responsible for CIT biosynthesis.

This Special Issue contains 13 review and research articles concerning *Monascus* fungi and their related products. The review article titled “From Random Perturbation to Precise Targeting: A Comprehensive Review of Methods for Studying Gene Function in *Monascus* Species” [Contribution 1] systematically examines the roles of various genetic manipulation techniques in advancing the molecular biology of *Monascus* fungi and offers an outlook on the application of artificial intelligence (AI) technologies in analyzing and predicting *Monascus* metabolites and metabolic pathways, as well as in the development of new *Monascus*-related products.

This Special Issue contains six research papers focusing on improving the yield of MPs—a group of representative secondary metabolites produced by *Monascus* fungi—as well as controlling citrinin production. The paper titled “Mutation Breeding of *Monascus* to Produce a High Yield of Orange Pigment and Low Citrinin Content Using the ARTP Method” [Contribution 2] describes a mutation breeding approach employing the ARTP method to enhance the high-yield production of *Monascus* yellow pigment while simultaneously reducing citrinin content. In the study “Transcriptome Analysis Revealed the Molecular Mechanism of Acetic Acid Increasing *Monascus* Pigment Production in *Monascus ruber* CICC41233” [Contribution 3], the molecular mechanisms by which acetic acid enhances MPs production in *Monascus ruber* CICC41233 are explored based on transcriptome analysis results. In the study “Co-Culture of *Monascus purpureus* and *Aspergillus niger* Isolated from Wuyi Hongqu to Enhance *Monascus* Pigment Production While Inhibiting Citrinin Production” [Contribution 4], the mechanism by which the co-culture of *M. purpureus* and *A. niger* isolated from Wuyi Hongqu enhances MPs production while inhibiting CIT production is investigated. In the study titled “Mechanistic Insights from Transcriptomics: How the Glucose Transporter *gltp*1 Gene Knockout Enhances *Monascus* Pigment Biosynthesis in *M. ruber* CICC41233” [Contribution 5], the authors utilize transcriptomic analysis results to elucidate the mechanism by which knockout of the glucose transporter *gltp*1 gene enhances MPs synthesis. In the study “Enhancement of *Monascus* Azaphilone Pigments Production Without Citrinin Contamination by Targeting Overexpression of Histone Acetyltransferase *MrEsa*1 and Deletion of Polyketide Synthase PksCT” [Contribution 6], a strain capable of producing no CIT is obtained by simultaneously knocking out the CIT polyketide synthase gene *pksCT* and overexpressing the histone acetyltransferase gene *MrEsa*1. The study titled “Role of *IscA*1 in Low-Frequency Magnetic Field-Associated Changes in *Monascus* Pigments and Citrinin Levels in *Monascus purpureus*” [Contribution 7] investigated the role of the *IscA*1 gene in the production of MPs and CIT by *M. purpureus* under low-frequency magnetic field conditions.

Three research papers are published in this Issue addressing the improvement in the yield of MK, another representative secondary metabolite produced by *Monascus* species. In the paper titled “Improvement of Monacolin K and Pigment Production in *Monascus* by 5-Azacytidine” [Contribution 8], it is found that the addition of 5-azacytidine to the culture medium can enhance the yields of both MK and MPs. In the study “Optimization of Co-Culture Between *Monascus purpureus* and *Saccharomyces cerevisiae* on Selenium-Enriched *Lentinus edodes* for Increased Monacolin K Production” [Contribution 9], by optimizing the co-culture conditions for *M. purpureus* and *S. cerevisiae* in the culture medium of selenium-enriched *Lentinus edodes* fruiting bodies, a fermentation product with high MK yield and elevated selenium content was obtained. In the study “Molecular Mechanism of *Mok I* Gene Overexpression in Enhancing Monacolin K Production in *Monascus pilosus*” [Contribution 10], the molecular mechanisms underlying the enhancement of MK production by *M. pilosus* through *Mok I* gene overexpression are investigated.

In addition, in this Issue, the study titled “Comparative Effects of Monascin and Monascinol Produced by *Monascus pilosus* SWM-008 on Pro-Inflammatory Factors and Histopathological Alterations in Liver and Kidney Tissues in a Streptozotocin–Nicotinamide-Induced Rat Model” [Contribution 11] compares the effects of monascin and monascinol—produced by *M. pilosus* SWM-008—on pro-inflammatory factors and histopathological alterations in the liver and kidney tissues of a streptozotocin–nicotinamide-induced rat model. The paper “Hydroxyl Radical Formation: A Key Mechanism and Regulatory Target in Monascin Photo-Degradation” [Contribution 12] analyzes the formation and regulatory mechanisms of the hydroxyl radical, a key factor driving the photo-degradation of monascin. The paper “Characterization of Key Metabolic Markers in Hongqujiu Across Different Aging Years Using Metabolomics” [Contribution 13] employs metabolomics techniques to analyze and compare key marker compounds in Hongqujiu wines aged for different periods of time.

A total of 13 papers are collected in this Isse on *Monascus* spp. and their products, all authored by scholars from the Mainland and Taiwan of China. This reflects that Chinese researchers are significantly ahead of their international counterparts in the study of *Monascus* and their metabolites, as well as in the development of related products. This is due to the fact that currently only China and other Asian countries such as Japan produce and consume products derived from *Monascus* spp., resulting in limited research on *Monascus* spp. and related products being conducted by scholars from other countries. Nevertheless, some international scholars are also engaged in research related to *Monascus* spp. Among them, the research group led by Professor Petra Patakova from the Czech Republic has conducted systematic studies on *Monascus* spp. Additionally, Brazilian scholars have also contributed to the classification of *Monascus* spp.

In conclusion, Chinese scholars have conducted systematic research on the molecular biology of *Monascus* spp. and their product development, obtaining many encouraging results. *Monascus* spp. may be one of the edible filamentous fungi that Chinese researchers have studied most comprehensively and systematically to date. Although some Chinese scholars have undertaken considerable work on the taxonomy of *Monascus* spp., for various reasons, the relevant research findings have not yet been published in internationally recognized academic journals.

## Data Availability

No new data were created or analyzed in this study. Data sharing is not applicable to this article.

